# Objective health literacy skills among ninth graders in Finland: outcomes from a national learning assessment

**DOI:** 10.1177/14034948211019798

**Published:** 2021-06-14

**Authors:** Anna-Mari Summanen, Juhani Rautopuro, Lasse K. Kannas, Leena T. Paakkari

**Affiliations:** 1 Faculty of Sport and Health Sciences, University of Jyväskylä, Finland; 2 Finnish Institute for Educational Research, University of Jyväskylä, Finland

**Keywords:** Health literacy, pupils, school, objective health literacy, curriculum

## Abstract

**Background:**

Health literacy (HL) is an important determinant for maintaining and improving health throughout the lifespan. This underlines the need to monitor HL, including among adolescents, and to understand the factors explaining HL, with a view to decreasing differences in HL. The aim of this study was to objectively measure HL, and the relationship between HL and socio-demographic factors (gender, language of instruction, pupils’ educational aspirations, parents’ educational background and pupils’ school achievement) among pupils (*n* = 3652) at the end of basic education in Finland.

**Methods:**

A nationally representative assessment, which included 55 items on HL, was conducted as a traditional paper-and-pencil test in schools. The data were analysed via descriptive statistics and a two-level hierarchical linear model to determine how the socio-demographic factors affected HL.

**Results:**

The pupils’ average score on the HL test items was 58.9%, indicating a *satisfactory* HL level. A two-level hierarchical linear model showed that the variables (gender, language of instruction, pupils’ educational aspirations, parents’ educational background and pupils’ school achievement) predicting the HL level had statistically significant effects.

**Conclusions:**

Among ninth graders (15–16 years old), almost one third of the boys and 8% of the girls exhibited an unsatisfactory HL level. The study also confirmed the importance of school-related factors in explaining HL differences. Since low HL has been linked to several unfavourable health indicators and has been viewed as an underestimated problem in times of pandemic and other health crises, the findings suggest that the HL level of Finnish ninth graders is a clear public-health issue.

## Background

Health literacy (HL) has received increasing attention as an important determinant of health behaviour and health, and as a means to promote health in different age groups [1,2]. Health literacy has been defined and conceptualized in multiple ways [3]. One well-known definition is that of the World Health Organization (WHO) [4]: ‘Health literacy describes the cognitive and social skills which determine the motivation and ability of individuals to gain access to, understand and use information in ways which promote and maintain health.’ In the present study we used the HL definition of Paakkari and Paakkari [5], who defined HL as consisting of five core components, namely theoretical knowledge, practical knowledge, critical thinking, self-awareness and citizenship. This definition has been found to be well-suited to school contexts.

The COVID-19 pandemic has shown low HL to be an underestimated problem, and HL has emerged as an important means to prevent both communicable and non-communicable diseases globally [6]. In the Shanghai Declaration on Health Promotion [7] and subsequently via the WHO European Roadmap for Implementation of HL [8], the WHO has established a strong global and regional mandate for public policy on HL.

According to WHO, HL forms an essential part of the skills and competencies that are developed over a lifetime, and should be advanced, especially through the school curriculum [7]. Finland is one of the few countries in which HL has been explicitly used as a framework for identifying and describing health education (HE) learning goals within the national core curriculum. Moreover, since 2001, HE has been a statutory, stand-alone and obligatory school subject in Finnish basic education and upper secondary education. There is now a clear and explicit emphasis on HL in the core curriculum (introduced in schools in 2016). The overall aim of HE as a school subject is to develop health-literate pupils [9].

The measurement of HL is a rapidly developing area among school-age children [10–12]. This can partly be explained by findings indicating that HL serves as an independent factor to explain disparities in health (i.e. the lower the HL, the poorer the health), including among adolescents [13–15]. Moreover, children’s health and differences in health status track forward to adulthood [16]. Among adolescents, low HL has been explained via various socio-demographic factors, including age [11], gender, parents’ education and family income [11,15]. There are further associations with non-academic educational aspiration, poorer school achievement, and learning difficulties [11].

Discussion of HL should not be limited merely to seeing low HL as a risk factor for poor health among adolescents. Indeed, HL should also be seen as an asset supporting the development of autonomy, empowerment [4] and the collective good [7]. It promotes an equitable distribution of power, including power over knowledge and knowing (involving for example a critical examination of universally accepted ‘good health practices’). Furthermore, it enables adolescents to find their own voice in the pursuit of freedom [17], highlighting the fact that the development of HL goes beyond health benefits alone. In addition, it is related to seeing adolescents as citizens in their own right, with their HL and health viewed as valuable *per se*, and not merely because these track forward to adulthood.

A view of HL as an asset [18] – and not just as a means of reducing health risks – was adopted into the Finnish national HE school curriculum in 2016. The inclusion is in line with Nutbeam [4], who emphasized the ‘asset’ concept of HL as applicable to both schools and healthcare settings. Within the HE school curriculum the definition of HL proposed by Paakkari and Paakkari has been applied, with HL being seen as composed of theoretical knowledge, practical knowledge, critical thinking, self-awareness and citizenship [5].

Previous studies on adolescents’ HL levels have mainly focused on the determination of perceived competence (as measured by subjective self-reporting) in various health-related issues (e.g. using HLSAC [12]). There is a lack of research applying an objective competency test to measure HL levels, and going beyond the mere measurement of functional literacy skills.

## Aims

In the present study, we explored the objectively measured HL of Finnish school-age pupils aged 15–16. The more specific research questions were: (a) what is the objectively measured HL level of school-age pupils in Finland at the end of basic education? (b) How do HL levels vary according to selected socio-demographic factors (gender, language of instruction, pupils’ educational aspirations, parents’ educational background, pupils’ school achievement)?

## Methods

### Participants and data collection

The data for this study were collected as part of a statutory national assessment of learning organized by The Finnish National Agency for Education (EDUFI) in 2013 [19]. The assessment constituted the first national examination of HE learning outcomes and of adolescents’ HL levels in Finland, applying an objective measure within a school HE context [19]. The aim of the assessment was to examine how well the aims of the national core curriculum in HE had been met, and to determine the overall national competence level in HE as a school subject, plus its core learning outcome, that is HL. This assessment took place under the Basic Education Act [20]. The assessment instrument was prepared on the basis of the goals, core content areas and criteria defined for a grade 8 level in HE (grade scale 4–10), in line with the national core curriculum for basic education [21], and the five core components of HL [5].

The data were collected via two-stage stratified random sampling. The first stage incorporated a sample of schools, which were regionally stratified prior to the sampling. In the second stage, a random sample of pupils was selected from each of the sampled schools. The sample reflected the structure of the population and was representative of Finnish pupils and their schools [19]. The response rate in the present study was 96%.

In total, 115 schools (90 Finnish-speaking schools, *n* = 3138 pupils and 15 Swedish-speaking schools, *n* = 514 pupils) were selected. The final sample size was 3652 pupils (girls, *n* = 1754; boys, *n* = 1898). The pupils were informed of the confidentiality of the data, and made aware that only group-level results would be reported at national level. The conduct of the tests followed the practices established by EDUFI [22].

### The objective measurement of HL

HL was measured using a broad 55-item paper-and-pencil test (37 multiple-choice questions, plus 18 open-ended questions). The test included different types of items with different difficulty levels, and covered the core content the HE curriculum, together with the various dimensions of HL. The HL levels were determined according to the number of correct solutions achieved by the pupils, converted to a percentage. The test formed an extensive, comprehensive and multidisciplinary instrument for measuring HL, and was found to have good internal consistency (reliability = 0.87). The measurement instrument is described in more detail in another article [23].

The test items were based on criteria that indicate good skills (corresponding to numerical grade 8) in the national core curriculum [21]. The standard deviation of the entire test was about 15 percentage points. The criteria for the levels were as follows: Poor HL (under 33.0%), Low HL (33.0–47.99%), Satisfactory HL (48.0–62.99%), Good HL (63.0–77.99%) and Excellent HL (over 78.0%). These classification terms were based on the terms used in Finnish schools.

### Socio-demographic variables

We further collected data on:

(a) gender;
(b) parents’ educational background (questions on whether the mother and father had completed the matriculation examination, options: yes/no/I don’t know);
(c) the pupil’s educational aspiration (What do you aspire to study after basic school? Options: I will go to general upper secondary school/I will go to a school of vocational education and training/I will acquire additional skills for studies after basic school/I will go to work / I will have a free year from studying); and
(d) the pupil’s school achievement (grades in HE, mother tongue and literature, and mathematics; grades 4–10).

In addition, the school language of instruction (Finnish or Swedish) was used as a background variable.

### Statistical analysis

The data were analysed via a range of well-established quantitative methods. In addition to the most common descriptive statistics (i.e. frequency distributions and measures of central tendency and variation), more sophisticated methods were applied. Due to the multistage sampling and hierarchical structure of the data, multilevel analysis was used to analyse group differences and to model the associations of background variables with HL. A mixed-methods model (also known as the random coefficient or hierarchical linear model) was applied [24]. This method makes it possible to satisfactorily estimate the standard errors in hierarchical datasets. The analyses were conducted using SPSS (version 24) and STATA software.

A large set of items was tested before the test [23]. The central orientation and variance were calculated for each item. The consistency of the assessment tasks was measured using Cronbach’s alpha coefficient [25]. In addition, the discrimination parameter and the difficulty parameter for each item were calculated via item response analysis (applying the generalized partial credit model) [26].

## Results

The average score in the test was 59% (SD = 14.8), which indicates satisfactory HL competence. Table I provides descriptive statistics of the students’ achievements according to some key background variables. The last column of the table shows the 95% confidence interval compared to the reference category. This interval takes into account the intra-class correlation presented in Table II.

**Table I.** The health literacy (HL) frequencies and mean scores (%) according to some key background variables.

Background variables	Frequencies (%)	Mean HL score (SD)	95% confidence interval (difference)
**Gender (*p* < 0.001)**			
Girls	1754 (48.0)	64.1 (13.3)	9.0–10.8
Boys (ref.)	1898 (52.0)	54.2 (14.6)	
**Language of instruction (*p* < 0.05)**			
Finnish	3138 (85.9)	59.3 (14.8)	0.3–6.3
Swedish (ref.)	514 (14.1)	56.5 (14.7)	
**Educational aspiration (*p* < 0.001)**			
School of vocational education and training (ref.)	1662 (45.5)	51.8 (13.8)	
General upper secondary school	1914 (52.4)	65.4 (12.4)	12.5–14.0
Other plans	29 (2.1)		
**Education of parents (*p* < 0.001)**			
Matriculation examination – both parents	659 (18.0)	64.2 (14.1)	6.1–8.8
Matriculation examination – one parent	1135 (31.2)	61.1 (14.2)	2.7–4.9
Matriculation examination – neither parent (ref.)	1155 (31.6)	57.4 (14.2)	
Missing data	703 (19.2)		
**Average grade of school subjects health education, maths, and mother tongue and literature (*p* < 0.001)**			
5 (ref.)	43 (1.2)	38.6 (11.9)	
6	400 (11.0)	45.3 (11.6)	2.4–8.8
7	898 (24.6)	51.8 (12.6)	8.3–11.2
8	1195 (32.6)	59.6 (12.1)	16.3–22.1
9	941 (25.8)	69.2 (10.5)	25.5–31.4
10	151 (4.1)	76.8 (11.6)	32.5–38.8
Missing data	24 (0.7)		

**Table II.** The results of the two-level hierarchical linear model (dependent variable: health literacy level).

	Null model	Model 1	Model 2	Model 3
**Independent variables**	Coef. (SE)	Coef. (SE)	Coef. (SE)	Coef. (SE)
**Constant**	58.9 (0.54)***	54.7 (0.60) ***	59.8 (0.81) ***	48.0 (5.51) ***
**Gender (ref. Boy)**				
Girl		9.9 (0.44) ***	6.9 (0.59) ***	3.6 (0.51) ***
**Language of instruction (ref. Finnish)**				
Swedish		−3.7 (1.52) *	−6.7 (1.44) ***	−4.9 (1.38) ***
**Parents’ education (ref. None matriculated)**				
One matriculated			1.9 (0.69) **	1.0 (0.58) n.s.
Both matriculated			4.4 (0.76) ***	1.5 (0.65) *
**Educational aspirations (ref. vocational education)**				
General upper secondary school			2.7 (0.65) ***	2.8 (0.54) ***
**School grade average (ref. 5)**				
6				−0.6 (5.8) n.s.
7				3.5 (5.5) n.s.
8				11.6 (5.5) *
9				19.1 (5.5) ***
10				26.6 (5.6) ***
**Variance components**				
Between schools	24.4	23.2	18.5	18.1
Within schools	195.5	171.1	123.1	85.9
Total variance	219.9	194.3	141.6	104.0
**Intra-class correlation**	0.11 (11%)	0.12 (12%)	0.13 (13%)	0.17 (17%)
**Variance explained (%)**				
Between schools		5.0	24.2	25.8
Within schools		12.5	37.0	56.1
Total variance		11.6	35.6	52.7

n.s. not significant; *p < 0.05; **p < 0.01; ***p < 0.001.

**Figure 1.**
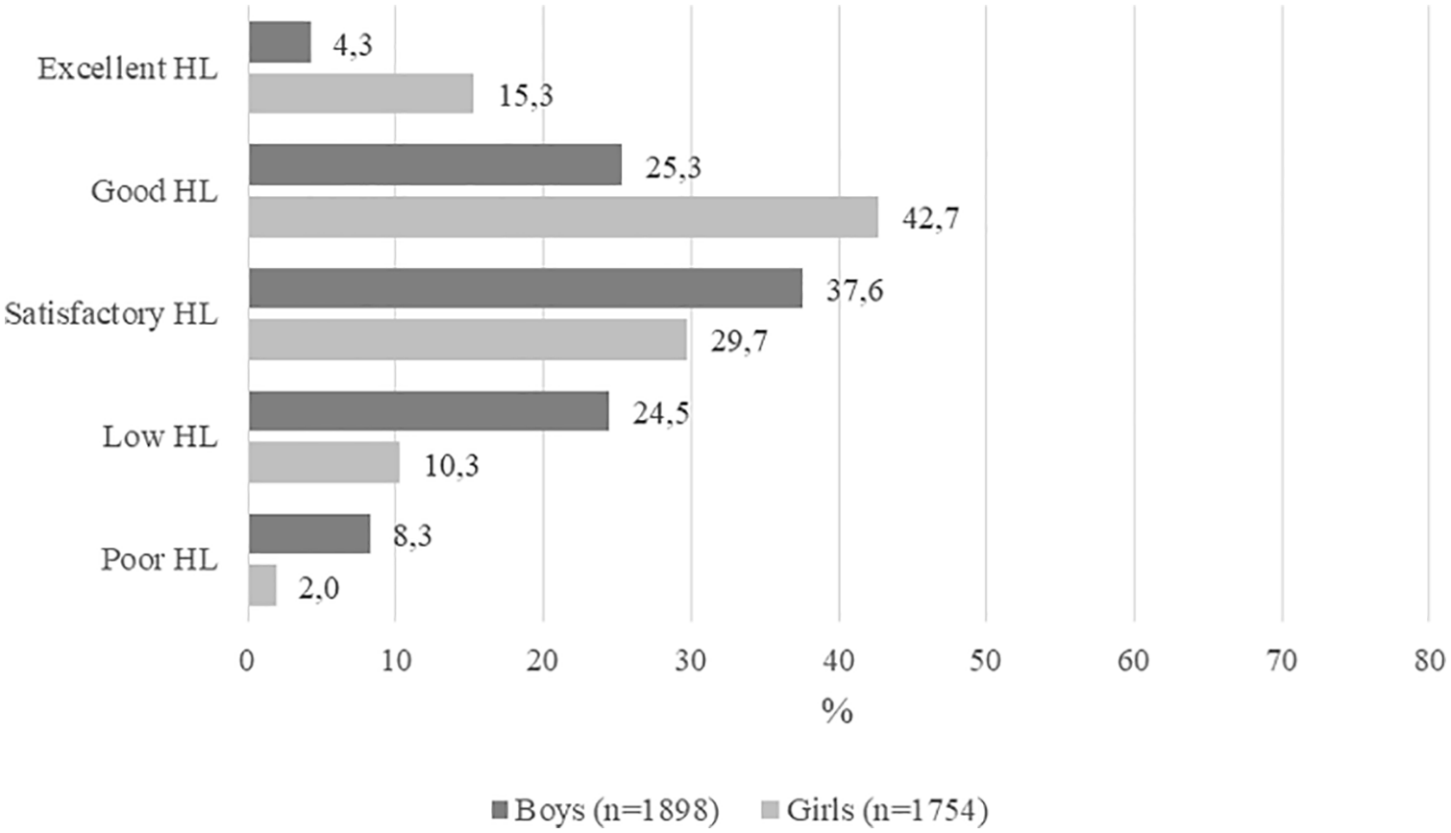
Health literacy (HL) levels among the pupils.

The average score for girls was about 64%, indicating good HL competence. The boys’ score (about 54%) was ten percentage points below that of the girls, indicating satisfactory HL competence. As evidenced in Figure 1, about 30% of the boys and 58% of the girls had a good HL level or higher. Almost one boy out of three (32.8%) but only 12% of the girls manifested poor or low HL competence.

We applied a two-level linear model taking into account the hierarchical structure of the data in order to predict HL achievement. The predictors were gender, language of instruction, pupils’ educational aspirations, parents’ educational background and pupils’ average school achievement (grades in HE, mathematics, and mother tongue and literature). The results are shown in Table II.

The first model (the null model) shows the variation between schools and within schools. The intra-class correlation (ICC) indicates that 11% of the variance in students’ achievement can be explained by school differences.

In Model 1, gender and the language of instruction in the school were added as fixed effect variables. They were chosen for the first model because they are essential factors when one considers the distribution of learning outcomes in Finland. This model shows that girls’ achievements are on average nearly 10% higher than those of boys (*p* < 0.001); moreover, the results in Swedish-speaking schools are almost 4% lower than those of Finnish-speaking schools (*p* < 0.05). These two variables explain 5% of the between-school variance and about 12% of the within-school variance presented in the null model.

In Model 2, the parents’ education and the students’ educational aspirations were added to the model. These variables were chosen because recent studies in Finland have highlighted their impact on pupils’ learning outcomes [27]. As Table II shows, the correspondence between parents’ education and learning outcomes is positive and statistically significant. In addition, students aiming at general upper secondary education perform better than those aiming at vocational education. When the effect of these variables is controlled, the gap between girls and boys is slightly smaller, but the gap between the language groups is larger. When these variables are taken into account, 24% of the between-school variance and 37% of the within-school variance presented in the null model can be explained.

Finally, the pupils’ average school grades were added to the model (Model 3). The reference category was school grade 5 (low). In pr actice, only students with very good and excellent school grades (9 and 10) had clearly better results. When the school grades were controlled, the gender gap became narrower, and the language gap was closer to Model 1. The final model explained 26% of the between-school variance and 56% of the within-school variance presented in the null model.

## Discussion

This study is the first to provide insights on objectively measured population-based HL levels among school-age children in Finland, and related socio-demographic factors. Clear associations were found between pupils’ HL and gender, the language of the school, pupils’ educational aspirations, parents’ educational background and pupils’ school achievement. The associations remained after all the factors were added to the same model.

Gender was clearly associated with HL: girls had significantly higher HL than boys. Previous results on the significance of gender in HL have varied. Some studies have shown that girls have higher HL [12], while in other studies boys have shown higher HL, or else no gender differences have been found [13]. It should be noted that most previous studies have focused on subjective HL. In the present objective HL study, the gender gap was greater than in previous (subjective) HL studies. Since studies conducted in Finland on the same age group have shown (a) no gender differences in subjectively measured HL levels, and (b) fairly high average subjective HL levels, our result here may indicate that pupils (especially boys) overestimate their HL competencies. Due to the various measures used, only tentative suggestions can be made on this aspect. Furthermore, the focus on subjective HL (i.e. “one’s perceived competence to pursue certain health-related courses of action”), describes another though important, part of HL [15]. Nevertheless the learning outcomes of teenagers in many school subjects in Finland have similarly indicated higher levels of excellence among girls [27]. For the sake of equality in HL and in education more broadly, it would be important to understand the factors underlying this phenomenon in efforts to decrease the gap between the genders. One explanatory factor might be that the overall subjective relevance of school is less for teenage boys than for girls [28]; this, in turn, would influence their weaker performance in this kind of broad-based test. Alternatively, the higher school achievement of girls might be due to stronger school motivation, higher verbal intelligence, greater conscientiousness, or stronger self-discipline [29]. Gender inequality is an issue for both educational policy and public health, and requires further research.

The language of instruction is an important background variable, given that Finland is a bilingual country with two official languages, Finnish and Swedish. For this reason, the language of instruction has been researched fairly frequently in relation to learning outcomes. In this study we found a significant association between HL and the language of instruction, with pupils in Finnish-language schools outperforming pupils in Swedish-speaking schools. This is consistent with findings on learning outcomes in several other school subjects in Finland [27]. Such a clear difference in HL performance is a key inequality issue, requiring more detailed investigation. Paradoxically, despite studies showing a clear association between higher HL and better health, Swedish speakers in Finland as a whole have better health than Finnish speakers [30]. To clarify the matter, research will have to take into account not only language groups, HL, and health indicators, but also broader environmental and cultural factors that may moderate and/or mediate the associations.

When we compared the pupils’ educational aspirations, we observed higher HL among those who, after basic education, planned to continue on an academic path (general upper secondary education), as compared to those who intended to pursue a non-academic path (vocational education and training). This confirms existing findings [14], given that previous studies have found a positive relationship between HL and the education level of both adolescents and parents, with higher educational levels predicting HL. In any case, our result here is in line with previous research on learning outcomes [27].

Among a substantial proportion of ninth graders (32.8% boys, 12% girls) the HL level was less than satisfactory. Low HL has been linked to several unfavourable health indicators [15] and noted as an underestimated problem in times of pandemic [6]. Thus, the findings from this study suggest that the HL level of Finnish ninth graders is a clear public health issue. The HL challenges were especially salient among pupils with poor school achievement, pupils seeking vocational education and training after basic school, and boys in general. These low-scoring pupils are at risk of falling irrecoverably behind in terms of knowledge, education and health, with implications for both public health and educational policy. A few years ago, HE as a stand-alone school subject was removed from vocational education and training in Finland. The present study suggests that precisely the opposite course of action should be followed, with efforts to strengthen the position of HE, both in basic education and in the upper secondary level of education. Moreover, the fact that HL can be developed and learned within HE [15], underlines the essential role of HE in reducing health inequalities.

HL requires a broad variety of learning opportunities, indicating that a range of growth environments should be involved in developing HL among children and young people [31]. In efforts to develop HL, it is increasingly important for people to have access to a variety of sources of information, so that they can make their own decisions on health topics. It is true that HE as a school subject is one way to construct and improve pupils’ HL; nevertheless, other contexts (including sport clubs, healthcare systems and youth organizations) are also needed [32]. In addition, it is necessary to support parental HL. Not everything can be made the schools’ responsibility, and a holistic and multidisciplinary partnership is needed in different sectors of society to improve adolescents’ HL.

One limitation of the present study relates to the questions on parents’ educational background. The matriculation examination provides a measure of the parents’ educational level on the learning outcomes evaluated by EDUFI. However, ‘parents’ matriculation examination’ can be a difficult concept for school-age children. They may not have information on the parents’ educational background. This view is partly supported by the large amount of missing data (19.2%) on this issue in the present study.

Following the Shanghai Declaration [7], many countries and governments around the world developed national policies, programmes, strategies and targets to improve HL in their populations [33]. Finland made important decisions on this matter even before that declaration. The setting up of HE as a school subject, including HL theory, is one example of this. Childhood and adolescence form pivotal periods in which to instil an HL lifestyle in daily life and to promote HL; these in turn can result in healthier lifestyles and better long-term health outcomes for adolescents. Schools have an important role in enhancing pupils’ knowledge, skills, critical thinking and citizenship with regard to health issues, and in improving their ability to make choices on health and wellbeing. In future, we intend to research the relationship between pupils’ objective and subjective HL, and also the ways in which objective HL is related to the teaching methods applied.
